# The Loss and Gain of Functional Amino Acid Residues Is a Common Mechanism Causing Human Inherited Disease

**DOI:** 10.1371/journal.pcbi.1005091

**Published:** 2016-08-26

**Authors:** Jose Lugo-Martinez, Vikas Pejaver, Kymberleigh A. Pagel, Shantanu Jain, Matthew Mort, David N. Cooper, Sean D. Mooney, Predrag Radivojac

**Affiliations:** 1 Department of Computer Science and Informatics, Indiana University, Bloomington, Indiana, United States of America; 2 Institute of Medical Genetics, School of Medicine, Cardiff University, Cardiff, United Kingdom; 3 Department of Biomedical Informatics and Medical Education, University of Washington, Seattle, Washington, United States of America; Rutgers University, UNITED STATES

## Abstract

Elucidating the precise molecular events altered by disease-causing genetic variants represents a major challenge in translational bioinformatics. To this end, many studies have investigated the structural and functional impact of amino acid substitutions. Most of these studies were however limited in scope to either individual molecular functions or were concerned with functional effects (e.g. deleterious vs. neutral) without specifically considering possible molecular alterations. The recent growth of structural, molecular and genetic data presents an opportunity for more comprehensive studies to consider the structural environment of a residue of interest, to hypothesize specific molecular effects of sequence variants and to statistically associate these effects with genetic disease. In this study, we analyzed data sets of disease-causing and putatively neutral human variants mapped to protein 3D structures as part of a systematic study of the loss and gain of various types of functional attribute potentially underlying pathogenic molecular alterations. We first propose a formal model to assess probabilistically function-impacting variants. We then develop an array of structure-based functional residue predictors, evaluate their performance, and use them to quantify the impact of disease-causing amino acid substitutions on catalytic activity, metal binding, macromolecular binding, ligand binding, allosteric regulation and post-translational modifications. We show that our methodology generates actionable biological hypotheses for up to 41% of disease-causing genetic variants mapped to protein structures suggesting that it can be reliably used to guide experimental validation. Our results suggest that a significant fraction of disease-causing human variants mapping to protein structures are function-altering both in the presence and absence of stability disruption.

## Author Summary

Identifying the molecular changes caused by mutations is a major challenge in understanding and treating human genetic disease. To address this problem, we have developed a wide range of profiling tools designed to predict specific types of functional site from protein 3D structures. We then apply these tools to data sets of inherited disease-associated and putatively neutral amino acid substitutions and estimate the relative contribution of the loss and gain of functional residues in disease. Our results suggest that alterations of molecular function are involved in a significant number of cases of human genetic disease and are over-represented as compared to putatively neutral variants. Additionally, we use experimental data to show that it is possible to computationally identify the loss of specific functional events in disease pathogenesis. Finally, our methodology can be used to reliably identify the potential molecular consequences of disease-causing genetic variants and hence prioritize experimental validation.

## Introduction

Spurred by the advances in DNA sequencing, the accumulation of human genetic variation (and with it amino acid substitution data) has over the past two decades been unprecedented. Multiple databases and resources now enumerate and annotate amino acid substitutions, their functional impact, and association with inherited disease [1–3]. However, to further our understanding of human genetic variation and its impact on disease, it is necessary to elucidate the associated molecular alterations [4–6]. Thus, the step of identifying the underlying molecular mechanisms constitutes a serious impediment to understanding and treating human disease.

A straightforward approach to integrating genetic and molecular data is to search databases for structural and functional annotations at the variation site or in the neighborhood of interest, and then provide both the possible and the likely effects of mutations on these annotations [7–12]. Although this approach is useful, its major limitation is its dependence on previously observed and curated functional information as well as our inability, except in limited cases [9, 10], to cover mutations that create functional residues. Furthermore, the deterministic nature of data integration does not easily lend itself to a principled strategy of prioritizing many of the possible molecular mechanisms based on their likelihood to impact clinical phenotype, especially when a variant resides in the neighborhood of the functional site.

A more comprehensive approach to analyzing the effects of amino acid substitutions involves the use of statistical inference methods that predict functional impact. Although there are many studies that have adopted this strategy using the data from protein sequence or structure [13], most methods make inferences without specifying which functional property has been impacted. Such an approach, however, is feasible if the methodology can be developed to predict a specific function, say a phosphorylation site or a catalytic residue, which is then applied to sequence variants [14–16]. Furthermore, these specific functional predictions can be integrated with general variant effect predictors to provide probabilistic estimates of molecular mechanisms of disease [17].

While many successful machine learning models can be made based on sequence information alone, structural information can provide additional benefits [18, 19]. This suggests that further improvements could be made if specific predictors of protein function could be integrated into this pipeline [20]. Wang and Moult published a seminal work on the impact of germline variants on protein function [7]. They searched the Protein Data Bank (PDB) and used homology modeling to obtain 3D structures of wild-type proteins as a means to characterize the structural and functional effects of both disease and neutral variants. They reported that the majority of disease-causing substitutions affect protein stability, whereas a relatively small proportion directly disrupt molecular function. By contrast, Sahni et al. observed a rather larger fraction of function-impacting variants in their experimental studies into the impact of variants on protein-protein and protein-DNA interactions [21]. Their work therefore challenges the traditional view of the dominance of structure-impacting changes. Finally, Steward et al. examined the structural, functional and physicochemical features of wild-type protein structures where disease-causing variants occur [22]. Unfortunately, the scope of these and several other studies was limited to characterizing the functional effects of amino acid substitutions across a handful of protein functions [23, 24]. There is therefore a need for large-scale studies that use statistical inference methods based on protein structure to explore the relative contributions made by disruption of functional sites in disease pathogenesis.

In this work, we carry out a systematic study of the alterations of specific functional sites as the underlying molecular mechanisms of disease over a data set comprising germline disease-causing amino acid substitutions mapped to protein 3D structures. In particular, we develop multiple structure-based functional residue predictors and assess the impact of disease-associated substitutions on catalytic residues, metal-binding sites, macromolecular binding sites, ligand-binding sites, allosteric sites and post-translational modification (PTM) sites. We then quantify the extent to which disruption or introduction of particular types of functional site accounts for the deleterious impact of amino acid substitutions. Our results provide evidence to support the view that the increased and decreased propensity of particular functional activities are common in human inherited disease.

## Materials and Methods

### Probabilistic model for alteration of residue function

For a given protein structure and a missense variant, we are interested in estimating whether a particular residue functionality, say *f*, has been impacted. To achieve this, we broadly distinguish between two scenarios resulting in alteration of function: (i) the mutation disrupts protein stability and subsequently impacts residue function and (ii) the mutation does not impact stability and structure yet still leads to an altered function as a consequence of modified functional propensity. The latter scenario might occur, for example, for tyrosine-to-phenylalanine substitutions that result in minimal structural changes, yet the mutation itself may have significant impact on protein phosphorylation and downstream events. This is because phenylalanine cannot be phosphorylated to subsequently create an SH2 binding site [25]. We informally refer to the events of increased or decreased functional propensity as gain and loss of functional activity.

We formalize this approach as follows. Let *x* be a collection of features that encodes a particular mutation in a protein structure and let *P*(loss of *f*|*x*) be the probability of the loss of residue function *f* consequent to mutation. Then, we can write

$$P\left(\text{loss of }f|x\right)=P\left(\text{loss of }f|S,x\right)P\left(S|x\right)\;+\;P\left(\text{loss of }f|\bar{S},x\right)P\left(\bar{S}|x\right),$$

where the event *S* indicates that protein stability is significantly changed and $\bar{S}$ indicates that the stability is not significantly changed; i.e., $P\left(S|x\right)=1-P\left(\bar{S}|x\right)$. This expression gives a probabilistic formulation that can be used to estimate loss of a specific function *f*, with an assumption of a dichotomized impact on protein stability. We use this simple approach in part because of the issues involved in obtaining large amounts of high-quality data to assess the impact of sequence variants on stability. We note, however, that the expression can be generalized to multiple groups of stability disruption and in the limit the sum would turn into an integral. Now we briefly discuss estimating *P*(*S*|*x*), *P*(loss of *f*|*S*, *x*), and $P(\text{loss of }f|\bar{S},x)$.

The posterior probability of stability disruption *P*(*S*|*x*) can be determined by developing a computational model given a representative data set of variants that significantly impact stability of the protein (both stabilizing and destabilizing mutations) and a representative set of variants that do not. The negative data set can also be substituted by a large representative set of variants for which the impact on stability is unknown [26, 27]. This formulation falls into the category of positive-unlabeled learning [28], a version of semi-supervised learning in which the set of negative examples is unavailable or ignored; e.g., because available negative examples are biased.

Next, we discuss estimating $P(\text{loss of }f|\bar{S},x)$; i.e., the probability of the loss of function given that there is no significant stability disruption. Let *x*′ be a collection of features that encodes the structural environment of a residue and let *f* be the specific residue function of interest; e.g., whether the residue is phosphorylated, DNA-binding, etc. Let $P(f|x_{wt}^{\prime })$ denote the probability that the residue at this site is functional in the wild-type protein and $P(f|x_{mt}^{\prime })$ the probability that the mutated residue, at the same locus in the protein, is functional. We then define the probability of the loss of residue function *f* as

$$P\left(\text{loss of }f|\bar{S},x\right)=P\left(f|x_{wt}^{\prime }\right)\cdot \left(1-P\left(f|x_{mt}^{\prime }\right)\right),$$

where $1-P(f|x_{mt}^{\prime })$ gives the probability that the residue in the mutant protein is not functional. To estimate $P(\text{loss of }f|\bar{S},x)$, we can employ the same functional residue predictor to compute prediction scores on the wild-type and mutant proteins. That is, because there are no structural changes, the same structure-based classifier can be used to compute both $P(f|x_{wt}^{\prime })$ and $P(f|x_{mt}^{\prime })$, with the only difference being the replaced amino acid in the feature set $x_{mt}^{\prime }$. As before, the probabilistic model *P*(*f*|*x*′) can be developed using data sets of positive and negative examples or, in the absence of representative negative examples, using positive and unlabeled data. We will discuss the details of approximating the posterior probability of protein function in the next section. Finally, we can consider that large changes in protein stability and structure always abolish the function of a residue; i.e., $P(f|x_{mt}^{\prime })\approx 0$. This implies that

$$P\left(\text{loss of }f|S,x\right)\approx P\left(f|x_{wt}^{\prime }\right),$$

that is, the probability of the loss of function at a particular residue is roughly equal to the probability that the residue was functional in the first place.

In addition to the loss of protein function, we can also consider the event of the gain of residue function, where

$$P\left(\text{gain of }f|\bar{S},x\right)=\left(1-P\left(f|x_{wt}\right)\right)\cdot P\left(f|x_{mt}\right).$$

This formulation accounts for the changes in residue microenvironments that increase its functional propensity. While most amino acid substitutions found in nature are neutral or disruptive, there are many examples in which they lead to the gain-of-function events. For example, generation of a sequence motif NX[S/T] has been observed to result in gain of N-linked glycosylation events and disease [9]. Similarly, changes in catalytic residues have been observed to increase the efficiency of catalysis, also with phenotypic implications [29]. Furthermore, assuming that significant stability changes rarely lead to gain of function we can simply take

P(gain of f|S,x)≈0.

In this paper, we are interested in specific types of residue function *f* for which sufficiently large data sets could be extracted from biological databases. We organize these residues into catalytic residues, metal-binding residues, macromolecule-binding residues, ligand-binding residues, post-translationally modified sites, and allosteric residues. For the purposes of our study, we consider certain types of residues to be functional although they may also be important for protein stability. For example, a disruption of certain metal-binding sites, say a Zn^2+^-binding residue, will be considered here as disruption of functional residues that consequently impacts protein stability. We do, however, note that this distinction is somewhat philosophical.

### Training stability predictors and functional residue predictors

All classification models in this work were trained using the positive-unlabeled framework in which we are given a set of positive examples and a set of unlabeled examples. In the case of stability predictors, *P*(*S*|*x*), the positive examples represent mutations that have been experimentally shown to significantly impact protein stability; based on the previous studies we selected these mutations to be either stabilizing or destabilizing with |ΔΔ*G*| > 0.5 kcal/mol [30], although some other studies use higher values [31, 32]. The set of unlabeled examples, on the other hand, was selected using a database of human variants, dbSNP, mapped to available protein structures in PDB. In the case of functional residue predictors *P*(*f*|*x*′), the positive examples were selected by integrating structural and molecular data that provide experimentally observed functional residues, whereas the unlabeled examples were selected from a set of monomeric proteins in PDB. We will describe all data sets precisely at the end of the Methods section.

We next discuss how to train a classification model from positive and unlabeled data. Let $D_{L}={\left\{\left(x_{i},y_{i}\right)\right\}}_{i=1}^{m}$ be a labeled data set, where $x_{i}\in X$ is an input example and *y*_*i*_ ∈ {−1, +1} is its class label. Let $D_{U}={\left\{x_{i}\right\}}_{i=1}^{n}$ be a set of unlabeled examples. In the problem of learning whether a mutation impacts stability, *x* encodes a set of features corresponding to the mutation and *y* = +1 indicates large stability disruption. Similarly, in the case of functional site predictors, *x* encodes a particular residue microenvironment in a protein and *y* = +1 indicates that the residue is functional. In the positive-unlabeled formulation, all examples in $D_{L}$ have positive class labels, whereas $D_{U}$ is a mixture of positive and negative examples. The probability of positive examples *P*(*y* = +1) in the unlabeled set is referred to as the class prior. The task of the predictor is to learn the probability *P*(*y* = +1|*x*) when provided data sets $D_{L}$ and $D_{U}$.

Unfortunately, learning *P*(*y* = +1|*x*) is not straightforward because the negative examples are not available. To address this problem we rely on the body of work in semi-supervised learning that decomposes the problem into the training of a non-traditional classifier [26]; i.e., a model that distinguishes between labeled and unlabeled data, and estimating the class prior *P*(*y* = +1). We denote the posterior probability from a non-traditional classifier as *P*(*l* = +1|*x*), where *l* = +1 refers to the event of data point being labeled. We approximate these probabilities using kernel-based learning with support vector machine (SVM) classifiers as underlying optimization engines. Additionally, we estimate *P*(*y* = +1) using the AlphaMax algorithm [27], and point out other available options for an interested reader [26, 33].

Under mild assumptions [27], the output of a non-traditional classifier *P*(*l* = +1|*x*) can be converted into the output of a traditional classifier *P*(*y* = +1|*x*) using

$$P\left(y=+1|x\right)=P\left(y=+1\right)\cdot \frac{n}{m}\cdot \frac{P(l=+1|x)}{1-P(l=+1|x)},$$

where *m* and *n* are the sizes of labeled and unlabeled data sets, respectively. This predictor can now be applied to any data set D to compute the frequency of the phenomenon using the empirical mean formula $\frac{1}{\left|D\right|}\sum_{x\in D}P\left(y=+1|x\right)$.

### The probability of alteration for multiple types of function

We previously considered the loss and gain of the specific function *f* at a particular residue of interest. We now extend this definition to multiple types of functional residues as follows. Consider an event of loss of any function *f* from a set F. We can use previous reasoning to re-write the earlier expression as

$$P\left(\text{loss of }F|x\right)=P\left(\text{loss of }F|S,x\right)P\left(S|x\right)+P\left(\text{loss of }F|\bar{S},x\right)P\left(\bar{S}|x\right).$$

To compute this probability, we need to compute probabilities P(loss of F|S,x) and $P(\text{loss of }F|\bar{S},x)$. Because the functional data is too sparse to learn the joint (posterior) models of residue function, we consider two models to approximate this probability using the marginal (posterior) models that the residue is functional. In the first model that we refer to as the independence model, we consider each type of functional residue to be independent of others and write

$$P\left(\text{loss of }F|\bar{S},x\right)=1-\prod_{f\in F}\left(1-P\left(\text{loss of }f|\bar{S},x\right)\right).$$

The expression above is the probability that at least one of the functions from F has been lost. Because the functions are not in reality independent, this model may lead to overestimation. The second, more conservative model, approximates the probability of loss as

$$P\left(\text{loss of }F|\bar{S},x\right)=\max_{f\in F}\left\{P(\text{loss}\text{of}f|\bar{S},x)\right\}.$$

We refer to this model as the max model. Equivalent expressions can be written for P(loss of F|S,x) as well as for the gain-of-function events. We note that F may contain particular groups of functions, say all types of metal binding, or can be used for all functions considered in this work.

### Graphlet kernels

In this section we briefly summarize the graphlet kernel prediction framework and show how these kernels were used to train both stability predictors and functional site predictors.

#### Graphs

A graph *G* is a pair (*V*, *E*), where *V* is a set of vertices (nodes) and *E* ⊆ *V* × *V* is a set of edges. In a vertex-labeled (colored) graph, a labeling function *g* is defined as *g*: *V* → Σ, where Σ is a finite alphabet, commonly referred to as vertex alphabet. A graph without self-loops, i.e. where (*v*, *v*) ∉ *E*, ∀*v* ∈ *V*, is said to be simple. An undirected graph is a graph where the order of the vertices in each pair (*u*, *v*) ∈ *E* can be ignored; otherwise, the graph is said to be a directed graph. A rooted graph *G* is a graph together with a distinguished vertex termed the root.

#### Graphlets

A graphlet is a small, simple, connected, rooted graph. We refer to a graphlet with *n* vertices as an *n*-graphlet. For more information on graphlets, we direct the reader to [34–38].

#### Edit distance graphlet kernels

Consider a vertex-labeled graph *G* = (*V*, *E*, *g*, Σ), where |Σ| ≥ 1. Lugo-Martinez and Radivojac [38] defined the *m*-edit distance representation of vertex *v* as

$$\phi_{\left(n,m\right)}\left(v\right)=\left(\psi_{\left(n_{1},m\right)}\left(v\right),\psi_{\left(n_{2},m\right)}\left(v\right),\ldots ,\psi_{\left(n_{\kappa (n,\Sigma )},m\right)}\left(v\right)\right),$$

where

$$\psi_{\left(n_{i},m\right)}\left(v\right)=\sum_{n_{j}\in E\left(n_{i},m\right)}w\left(n_{i},n_{j}\right)\cdot \varphi_{n_{j}}\left(v\right).$$

In the previous expression $\varphi_{n_{j}}(v)$ is the count of the *j*-th labeled *n*-graphlet rooted at *v*, *κ*(*n*, Σ) is the total number of vertex-labeled *n*-graphlets and *E*(*n*_*i*_, *m*) is a set of *n*-graphlets such that for each *n*_*j*_ ∈ *E*(*n*_*i*_, *m*) there exists an edit distance path of length at most *m* that transforms *n*_*i*_ into *n*_*j*_. That is, the number of edit operations necessary to transform *n*_*i*_ into *n*_*j*_ is at most *m*, where edit operations are defined as insertion or deletion of vertices and edges, or in the case of labeled graphs, substitutions of vertex and edge labels. Finally, weights *w*(*n*_*i*_, *n*_*j*_) ≥ 0 are used to adjust the influence of pseudo counts and control computational complexity; in this study, we set *w*(*n*_*i*_, *n*_*j*_) = 1 if *n*_*j*_ ∈ *E*(*n*_*i*_, *m*) and *w*(*n*_*i*_, *n*_*j*_) = 0 otherwise.

The length-*m* edit distance *n*-graphlet kernel *k*_(*n*,*m*)_(*u*, *v*) between vertices *u* and *v* can be computed as an inner product between the respective count vectors *ϕ*_(*n*,*m*)_(*u*) and *ϕ*_(*n*,*m*)_(*v*). Hence, the length-*m* edit distance graphlet kernel function can be expressed as

$$k_{m}\left(u,v\right)=\sum_{n=1}^{N}k_{\left(n,m\right)}\left(u,v\right),$$

where *N* is a small integer; typically defined up to *N* = 5 for undirected graphs. Additionally, one can define two subclasses of edit distance kernels referred to as (vertex) label-substitution *k*^*l*^ (only allows substitutions of vertex labels) and edge-indel kernels *k*^*e*^ (only allows insertion or deletion of edges). It is worth noting that if *m* = 0, then *k*_*m*_, $k_{m}^{l}$ and $k_{m}^{e}$ are all equivalent to the standard graphlet kernel on labeled graphs [37]. In this work, we only considered the normalized kernel calculated as

$$k\left(u,v\right)=\frac{k^{*}\left(u,v\right)}{\sqrt{k^{*}\left(u,u\right)\;k^{*}\left(v,v\right)}},$$

where k*(*u*, *v*) can be *k*_*m*_(*u*, *v*), $k_{m}^{l}\left(u,v\right)$, or $k_{m}^{e}\left(u,v\right)$. The normalized kernel has been previously shown to have favorable performance with respect to non-normalized kernels [37, 38].

#### Practical aspects of training

**Fig 1:**
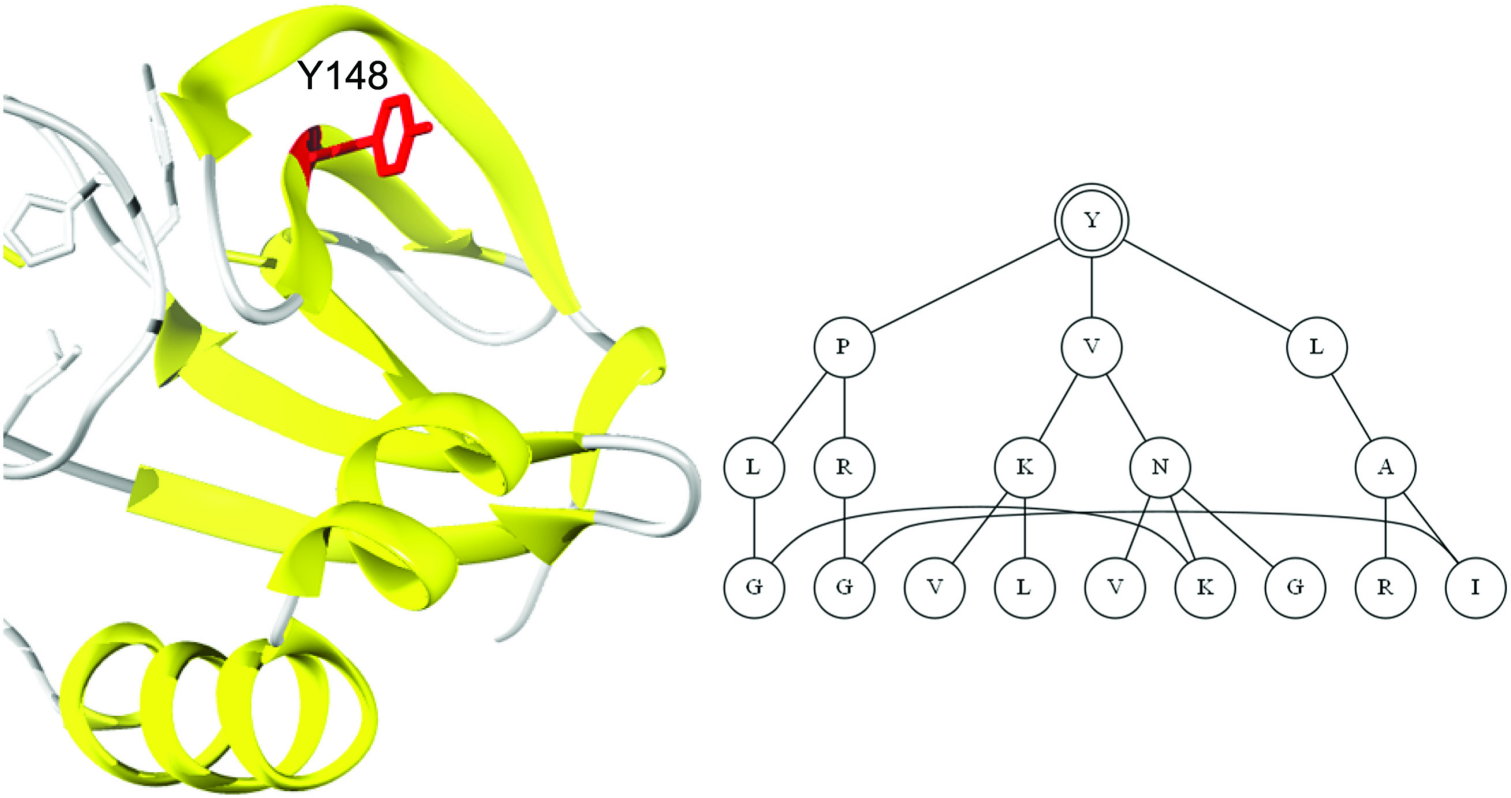
**Left: Structure of human aurora kinase A, chain A fragment (PDB entry 2j4z) with highlighted phosphorylation site Tyr148 (denoted as Y148).** Right: Corresponding level-3 protein contact graph centered at Tyr148 (denoted with double circles). Nodes represent amino acid residues and edges correspond to spatially neighboring residues (i.e. 6*Å* or less between *C_α_* atoms).

We used the graphlet kernel framework and SVM classifiers to construct all functional site predictors. First, we modeled protein structures as protein contact graphs, where each amino acid residue was represented as a vertex and two spatially close residues (i.e. 4.5*Å* or less between any two atoms) were linked by an undirected edge. Fig 1 illustrates a contact graph for a protein kinase rooted at a tyrosine residue at position 148. Next, we computed a set of normalized graphlet kernel matrices K using $k_{m}^{l}\left(x_{i},x_{j}\right),k_{m}^{e}\left(x_{i},x_{j}\right)$ and *k*_*m*_(*x*_*i*_, *x*_*j*_) for all pairs (*x*_*i*_, *x*_*j*_). For each k∈K, we used SVM^*light*^ [39] and the default value for the capacity parameter to train a predictor. We incorporated evolutionary information by extending the vertex alphabet Σ from the 20 standard amino acids to 40 based on the median residue conservation observed over the entire data set [38]. For example, the amino acid alanine was split into highly conserved alanines (represented as *A*) and other alanines (represented as *a*). Once each predictor was trained, we used Platt’s correction to adjust the outputs of the predictor to the 0-1 range [40].

In the case of stability predictors, we augmented the graphlet kernel representation using 33 features previously shown to be informative [41, 42]. In particular, we used 20 features for the 20 different amino acids to encode the mutation information (i.e. −1 for wild-type residue; +1 for mutated residue; 0 otherwise), and 13 features to encode the difference between physicochemical properties between wild-type and mutant amino acid residues [43–50].

### Evaluation of in silico predictions

The performance of each predictor was first evaluated through a per-chain 10-fold cross-validation. In each iteration of cross-validation, 10% of protein chains were selected for the test set, whereas the remaining 90% were used for training. This enforces that all data points from the same protein sequence belong to either training or test set and, thus, reduces the chance of overestimating the accuracy of the models. We estimated the area under the ROC curve (AUC), which plots the true positive rate as a function of the false positive rate and the Matthews correlation coefficient (MCC).

It is impractical to validate, in vitro or in vivo, the functional effects of each amino acid substitution in our data sets. Therefore, we used independent mutagenesis experimental data to additionally evaluate the performance of our functional site predictors and also evaluate predictions of the loss of functional residues. More specifically, we downloaded all human mutagenesis experimental data from UniProt as of September 2014. This data set comprised 14,933 substitutions from 3,044 distinct proteins. We removed all entries associated with more than one substitution. The resulting 11,425 sites were mapped to high-quality PDB structures using the same steps described in the next section. The final data set comprised 3,356 amino acid substitutions from 2,809 different sites in 880 proteins.

For each site in this data set, we extracted functional annotations related to metal binding, PTMs, active sites, macromolecular binding, ligand binding and allosteric activity. Then, for each functional site predictor, we built an independent test set such that (i) each site belonged to a chain that was less than 40% identical to any chain in the training data, (ii) there were at least five positive sites in each test set. The resulting set was used to assess the performance of the functional predictors independently of the cross-validation.

Similarly, we created a test data set to evaluate loss-of-function predictions as follows: we searched the description of the mutagenesis experiment such that there was an experimentally observed disruption of a functional site. The resulting non-redundant and filtered data sets were then used to estimate the AUC and MCC of all loss-of-function predictors. We attempted to carry out the same steps for gain-of-function events but there were insufficient data.

### Disease-associated mutation data

Missense variants causing inherited disease were obtained from the Human Gene Mutation Database (HGMD) as of June 2013. A set of unlabeled inherited variants was downloaded from dbSNP v.137. All amino acid substitutions were then mapped to protein structures in PDB as follows: (i) a database of amino acid sequences from X-ray crystallographic protein structures with more than 50 amino acids and resolution less than 2.5*Å* was created, and (ii) for each variant, a 51 residue long sequence centered around the wild-type amino acid at the variant position was aligned using BLAST [51] against the atom sequences in PDB. All alignments without an exact match; i.e., with gaps or sequence identity lower than 100%, were excluded from this study and in the case of multiple exact matches, the structure with the best resolution was selected. This resulted in 10,629 (out of 52,406) disease-causing amino acid substitutions and 8,417 (out of 282,625) unlabeled amino acid substitutions being successfully mapped to high-quality PDB structures. Table 1 summarizes both data sets. There exists an overlap between the HGMD (disease) and dbSNP (unlabeled) variants; the subset of dbSNP variants after the removal of HGMD variants will be referred to as putatively neutral variants (Table 1).

**Table 1:** Summary of amino acid substitution (AAS) data sets.

Data set name	*n*_AAS_	*n*_*s*_	*n*_PDB_	*n*_*c*_
Inherited disease	52406	10629	1177	1387
Unlabeled variants	282625	8417	3121	3585
Putatively neutral	282625	8049	3047	3500

For each data set, we show the total number of amino acid substitutions (*n*_AAS_), the number of substitutions mapped to PDB (*n*_*s*_), the number of PDB entries (*n*_PDB_) and the number of protein chains (*n*_*c*_).

### Functional site data sets

Metal ions annotated in X-ray structures from PDB as of May 2012 were selected using the HETATM field [52]. A metal-binding residue was defined as the residue that has at least one heavy atom (N, O or S) within 3*Å* of the metal ion. In order to build an unbiased classifier, we removed chains with: (i) more than 40% sequence identity with any other chain in the data set, (ii) crystallographic resolution greater than or equal to 2.5*Å*, and (iii) R-values greater than or equal to 30%. We only considered data sets with more than 100 metal-binding residues and we refer to these residues as positives. N-linked glycosylation sites were parsed from PDB and filtered in the same way as the metal-binding sites. In the case of phosphorylation sites, we used the data set assembled previously [20]. Catalytic residues were collected from the Catalytic Site Atlas v2.2.10 [53] and only literature-supported sites were kept as positive examples. As with the previous data sets, we filtered out chains with more than 40% sequence identity. DNA-binding sites were collected from Yan et al. [54], RNA-binding sites were downloaded from ccPDB [55] and protein-protein interaction (PPI) sites were obtained from Chung et al. [56]. Each data set was further filtered to remove redundancy. Protein-protein interaction hot spot residues were obtained from Lise et al. [57]. This data set was created from ASEdb [58] and only sites that mapped to PDB were used. Chains with more than 35% sequence identity were filtered out in the original work. In our analysis, we used ΔΔ*G* greater than 1kcal/mol as the cutoff for hot spots. Ligand binding data sets were collected from ccPDB as of November 2014 and allosteric site data were downloaded from ASD v2.0 [59]. For each data set, we removed chains with resolution greater than or equal to 2.5*Å*. Protein stability data was collected from Capriotti et al. [41]. We used the S1615 data set which consists of 1615 single site mutations extracted from 42 different proteins in the ProTherm database [60]. The attributes for each data point included solvent accessibility, pH value, temperature and energy change ΔΔ*G*. As previously noted, positive data points comprised mutations with |ΔΔ*G*|>0.5. We further filtered out 112 redundant data points.

Table 2 summarizes the protein stability and functional site data sets used in this study. In all situations, the unlabeled data set was constructed using a random sample of 10,000 residues selected from the 40% non-redundant set of monomers in PDB. This set was modified in the case of post-translational modifications to include only modifiable residues; e.g., Asn for N-linked glycosylation and Ser/Thr/Tyr for phosphorylation. It is important to emphasize that the set of negative examples was allowed to contain both buried and surface-exposed residues resulting in somewhat easier downstream classification problems. On the other hand, it allowed us to apply our methods to all PDB-mappable amino acid substitutions and make unbiased inferences related to different data sets.

**Table 2:** Performance assessment of structural and functional residue predictors using cross-validation on positive-unlabeled data sets.

Category	Site type	*n*_*c*_	*n*_+_	AUC	*sn*	*sp*	MCC
Protein stability	Stability (S)	40	1041	0.735	0.118	0.989	0.223
Metal binding	Calcium (Ca)	1092	4860	0.895	0.448	0.986	0.561
	Cadmium (Cd)	199	1003	0.905	0.233	0.986	0.350
	Cobalt (Co)	156	532	0.934	0.596	0.986	0.623
	Copper (Cu)	105	440	0.951	0.780	0.985	0.727
	Iron (Fe)	187	785	0.976	0.875	0.986	0.843
	Potassium (K)	287	978	0.679	0.129	0.986	0.211
	Magnesium (Mg)	1348	3282	0.859	0.435	0.986	0.561
	Manganese (Mn)	366	1344	0.945	0.665	0.985	0.727
	Sodium (Na)	961	2753	0.671	0.105	0.987	0.211
	Nickel (Ni)	254	680	0.932	0.565	0.986	0.621
	Zinc (Zn)	1307	5778	0.966	0.623	0.987	0.691
PTMs	N-glycosylation (Nglyco)	339	736	0.785	0.120	0.986	0.183
	Phosphorylation (Phos)	655	1157	0.810	0.375	0.987	0.504
Catalytic activity	Catalytic (Cat)	721	2224	0.934	0.433	0.985	0.561
Macromolecular binding	DNA-binding (DNA)	139	3791	0.815	0.193	0.987	0.332
	RNA-binding (RNA)	83	3436	0.783	0.187	0.985	0.319
	Protein-protein interaction (PPI)	112	4350	0.807	0.091	0.987	0.191
	PPI hot spots (Hotspot)	35	165	0.803	0.309	0.986	0.278
Ligand binding	ADP	162	2589	0.842	0.335	0.985	0.475
	ATP	104	1733	0.813	0.242	0.986	0.382
	FAD	80	2248	0.840	0.307	0.985	0.448
	FMN	42	788	0.824	0.284	0.985	0.384
	GDP	45	593	0.843	0.433	0.985	0.502
	GTP	22	366	0.716	0.145	0.986	0.181
	HEM	83	2246	0.847	0.220	0.986	0.361
	NAD	73	1663	0.831	0.259	0.985	0.393
	PLP	34	477	0.916	0.505	0.986	0.543
	UDP	27	398	0.684	0.080	0.987	0.103
Allosteric regulation	Allosteric (Allo)	108	682	0.636	0.041	0.985	0.050

For each data set, we show the number of protein chains (*n*_*c*_) and the number of positive examples (*n*_+_). Additionally, we choose a score threshold corresponding to a specificity (*sp*) of 99% and report sensitivity (*sn*) and MCC at this threshold, as well as AUC. In each classification problem, the number of unlabeled examples was set to 10,000. S1 Table predictions lists the full name of each ligand code used. For the purposes of this work, structurally important amino acid residues such as specific metal ion binding residues were considered a part of the portfolio of available residue functions.

## Results

In this section we present the development of a stability model and a series of structure-based functional site predictors in order to examine the molecular effects of genetic variants. We evaluate the predictors through cross-validation and using an independent data set. We then summarize our results in relation to the functional impact of disease-causing substitutions and compare them to putatively neutral variants.

### Assessment of functional site predictors

All classifiers developed in this study were constructed using positive and unlabeled data summarized in Table 2. Their performance was estimated via per-chain 10-fold cross-validation and is also shown in Table 2. S2 Table further lists the parameters for the best-performing kernel matrix obtained from a grid search over K, |Σ| = {20, 40}, *m* = {0, 1} and *N* = {4, 5}. Each predictor performance was assessed by means of the area under the ROC curve (AUC), sensitivity (*sn*) at 99% level of specificity (*sp*), and the Matthews correlation coefficient (MCC). The majority of predictors (26 out of 30) show good performance (≥ 70% AUC); however, we observe that functions related to smaller interfaces such as metal ions and active sites exhibit higher performance than other functional predictors. This result is not unexpected because predictors of macromolecular binding would have benefited from incorporating higher-order structural signatures such as clefts and pockets [20].

We also use an independent data set to evaluate a subset of functional site predictors, as depicted in S3 Table. Interestingly, most predictors, except for macromolecular binding models show similar or improved performance (AUC) values compared to those reported from cross-validation (Table 2). Overall, despite the variability of performance accuracies, limited number of independent data sets and the relatively small size of the validation data, these results provide evidence that functional site predictions are of sufficient quality to identify possible molecular alterations resulting from specific missense mutations.

A literature survey suggests that our predictors perform well when compared to established structure-based methods. Extensive comparisons with other work are difficult and were beyond the scope of this study as our main goal was to probabilistically assess molecular mechanisms of disease. A set of predictors built using the same methodology was best suited to this task.

### Estimating prior and posterior probabilities

To use the formal framework laid out in the Methods section, it is important that all methods approximate posterior distributions. Using positive and unlabeled data, we have approached this problem in two steps: (i) by developing classifiers that discriminate between labeled and unlabeled data, and (ii) by estimating the class priors of the positive class in the unlabeled data [27].

Estimated class priors are a particularly useful by-product of learning posterior distributions. For the stability predictor, we estimate up to 13% of unlabeled variants to significantly impact stability using the AlphaMax algorithm [27]. When the stability model was applied to disease variants only, we estimate 14% of these variants to be impactful using the empirical mean formula. It should be noted that when the known disease variants were removed from the unlabeled data set, only 7% of the remaining variants were estimated to severely impact stability. In the case of functional predictors, we applied the AlphaMax algorithm using a set of positive variants and a set of 10,000 variants randomly sampled from a set of non-redundant monomers in PDB (S4 Table). In the case of catalytic residues, we estimate that up to 3% of PDB residues to be catalytic; however, about 5% of disease-causing and 2% of putatively neutral variants were estimated to be catalytic residues, etc. Overall, we generally observe a larger fraction of function-impacting variants in the disease-causing data set as compared with the putatively neutral variants.

### Applying loss and gain functional site predictors to human variants

We applied the structure-based predictors on both the wild-type and mutant structural environments as a means to identify and categorize the functional effects of amino acid substitutions causing inherited disease. The distribution of scores on the putatively neutral variants was used as an empirical null distribution. We then used a particular false positive rate (FPR) value to determine a prediction threshold at which to assess the fraction of disease mutations with loss or gain scores that are as high as or higher than the threshold.

**Fig 2:**
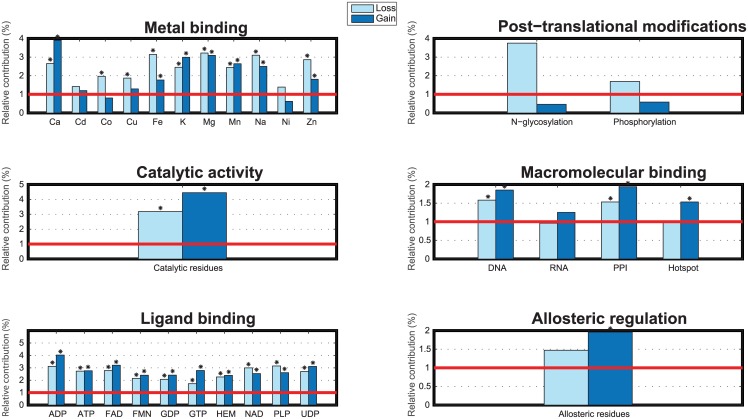
Percentage of disease variants with prediction scores at the 1% false positive rate threshold in putatively neutral variants. For each function *f*, the bars indicate the percentage (%) of disease mutations that have a greater $P(\text{loss}|\bar{S},x)$ and $P(\text{gain}|\bar{S},x)$ than a conservative threshold at 1% false positive rates (FPR). These thresholds are estimated from the null distributions of $P(\text{loss}|\bar{S},x)$ and $P(\text{gain}|\bar{S},x)$ on the set of dbSNP neutral data, respectively. *Indicates significant *p*-value measured as a one-tailed Fisher’s Exact test after Bonferroni correction for multiple hypothesis testing (*p* < 8.62 ⋅ 10^−4^). The red line indicates the percentage of neutral variants that have greater $P(\text{loss}|\bar{S},x)$ and $P(\text{gain}|\bar{S},x)$ which is exactly 1%.

Table 3 summarizes the relative contributions of disease mutations that either decrease (loss) or increase (gain) the propensity of functional sites at a conservative threshold of 1% FPR for six different prediction outputs. Fig 2 visualizes a subset of these results for the case when stability is not impacted. Together with Table 3, it provides evidence that the loss and gain of functional sites exist even when protein stability is not disrupted; e.g., in the case of loss of function see columns $P(\text{loss}|\bar{S},x)$ and $P(\text{loss},\bar{S}|x)$ that roughly have the same values as *P*(loss|*x*). We mention that when either a loss or a gain of function event is found to be statistically significant, the mutation of this type of functional residue is considered to be an active mechanism of genetic disease. For instance, at 1% FPR, we observe that loss of catalytic residues (Cat; 3.34%; *p*-value = 1.93 ⋅ 10^−28^) and iron-binding residues, (Fe; 3.17%; *p*-value = 2.06 ⋅ 10^−25^) are among the most significantly affected molecular mechanisms.

**Table 3:** Percentage of disease variants with prediction scores at the 1% false positive rate threshold in putatively neutral variants.

Data set	Single type loss events (%)			Single type gain events (%)		
	$P(\text{loss}|\bar{S},x)$	$P(\text{loss},\bar{S}|x)$	*P*(loss|*x*)	$P(\text{gain}|\bar{S},x)$	$P(\text{gain},\bar{S}|x)$	*P*(gain|*x*)
Ca	2.65*	2.35*	2.69*	3.90*	3.63*	3.63*
Cd	1.42	1.31	1.44	1.20	1.02	1.02
Co	1.97*	1.94*	1.98*	0.80	0.70	0.70
Cu	1.87*	1.82*	1.88*	1.29	1.15	1.15
Fe	3.14*	2.97*	3.17*	1.77*	1.62*	1.62*
K	2.45*	2.08*	2.45*	2.99*	2.60*	2.60*
Mg	3.22*	2.83*	3.14*	3.09*	2.89*	2.89*
Mn	2.42*	2.22*	2.45*	2.65*	2.47*	2.47*
Na	3.10*	2.55*	3.13*	2.50*	3.05*	3.05*
Ni	1.39	1.33	1.40	0.62	0.54	0.54
Zn	2.86*	2.64*	2.89*	1.81*	1.63*	1.63*
Nglyco	3.75	1.25	2.81	0.46	0.23	0.23
Phos	1.69	1.47	1.69	0.58	0.46	0.46
Cat	3.18*	2.90*	3.34*	4.45*	3.94*	3.94*
DNA	1.58*	1.39	1.61*	1.85*	1.75*	1.75*
RNA	0.96	0.89	0.96	1.25	1.05	1.05
PPI	1.53*	1.27	1.65*	1.94*	1.79*	1.79*
Hotspot	1.00	0.90	1.00	1.53*	1.45	1.45
ADP	3.12*	2.92*	3.16*	4.03*	3.68*	3.68*
ATP	2.73*	2.52*	2.76*	2.76*	2.41*	2.41*
FAD	2.77*	2.58*	2.81*	3.21*	2.92*	2.92*
FMN	2.15*	2.01*	2.17*	2.40*	2.18	2.18*
GDP	2.07*	1.99*	2.08*	2.41*	2.18*	2.18*
GTP	1.72*	1.48	1.72*	2.78*	2.15*	2.15*
HEM	2.26*	2.05*	2.35*	2.39*	2.05*	2.05*
NAD	3.00*	2.64*	3.09*	2.53*	2.15*	2.15*
PLP	3.15*	2.98*	3.10*	2.61*	2.37*	2.37*
UDP	2.70*	2.41*	2.71*	3.11*	2.46*	2.46*
Allo	1.47	1.24	1.49	1.96*	1.74*	1.74*
Model	Multi-type loss events (%)			Multi-type gain events (%)		
	$P(\text{loss}|\bar{S},x)$	$P(\text{loss},\bar{S}|x)$	*P*(loss|*x*)	$P(\text{gain}|\bar{S},x)$	$P(\text{gain},\bar{S}|x)$	*P*(gain|*x*)
Independence	3.71*	3.31*	3.89*	4.13*	3.37*	3.37*
Max	3.50*	1.93*	3.56*	4.35*	2.63*	2.63*

For each of the six prediction outputs and each function *f*, we show the percentage (%) of disease mutations that have a greater probability of loss and gain of function than a threshold corresponding to a 1% false positive rate (FPR). S1 and S2 Figs show an instance of the inverse cumulative distribution function of *P*(loss|*x*) and *P*(gain|*x*), respectively. These thresholds were estimated from the empirical null distributions of the probability of loss or gain of function on the set of dbSNP neutral data.

*Indicates significant *p*-value measured by a one-tailed Fisher’s exact test after Bonferroni correction for multiple comparisons. The *p*-value was separately estimated for each type of posterior distribution, jointly for loss and gain events ($p<\frac{0.05}{58}=8.62\cdot 10^{-4}$). The *p*-values for the combined models were corrected separately ($p<\frac{0.05}{4}=1.25\cdot 10^{-2}$).

Table 3 also summarizes the statistical enrichment of impact on at least one functional site from the entire repertoire of functions using the independence and max models (see Methods). Here we observe a strong enrichment in all categories of loss of function, with or without impact on stability, for both the independence and max models. Additionally, we also see an enrichment in the gain-of-function events. These results provide statistical support for many individual studies that identify loss of function as a signature of human inherited disease.

Overall, our results suggest that with some exceptions, the loss of functional residues is enriched and common in human inherited disease; similarly, the gain of functional residues is observed to be an active mechanism in catalytic activity, most types of ligand-binding residues, and majority of metal-binding residues. In contrast to previous studies, our results suggest that the loss and gain of PTM sites do not show statistically significant enrichment in disease (although we observe enrichment for the loss); however, we note that this may be due to a considerable reduction of training data imposed by the availability of protein 3D structures, especially given a relationship between post-translational modifications and intrinsically disordered proteins [61–64].

Table 4 shows the proportions of disease and putatively neutral variants across functional categories for which molecular mechanisms can be computationally hypothesized. In the first part of the table, we compute the fraction of variants for which exactly one of the member predictors reports a score as high or higher than the FPR-value determined threshold. These fractions were then computed separately for disease and neutral variants. For convenience, when a predictor outputs a value as high or higher than the value determined by a 1% FPR, we refer to this prediction as actionable hypothesis of loss or gain of function. On the other hand, when the FPR-based threshold is adjusted using the Bonferroni correction, we refer to these predictions are confident. For example, at a conservative *p*-value cutoff of *p* < 8.62 ⋅ 10^−4^, we find that 1.51% of mutations are likely to alter exactly one metal binding site and 1.43% may alter a single ligand binding site. For all groups of molecular mechanisms, we observe that the probability of observing a high alteration score is more than three times as likely as in the case of putatively neutral variants.

**Table 4:** Relative contribution of loss and gain of functional categories from amino acid substitutions.

Category	Loss (%)		Gain (%)		Loss or Gain (%)	
	Disease	Neutral	Disease	Neutral	Disease	Neutral
*I. Single mechanism*						
Confident biological hypotheses (*p*-value < 8.62 ⋅ 10^−4^)						
Metal binding	1.51	0.27	1.46	0.46	2.88	0.70
PTMs	0	0	0.01	0	0.01	0
Catalytic sites	0.73	0.06	0.40	0.07	1.14	0.14
Macromolecule binding	0.59	0.20	0.64	0.20	1.21	0.40
Ligand binding	1.43	0.47	1.86	0.46	3.07	0.87
Allosteric sites	0.09	0.06	0.25	0.06	0.35	0.12
All	3.03	0.84	3.29	0.97	5.80	1.70
Actionable biological hypotheses (*p*-value < 0.01)						
Metal binding	4.90	2.50	5.08	2.99	8.70	5.03
PTMs	0.30	0.17	0.08	0.22	0.39	0.40
Catalytic sites	3.34	1.00	3.94	1.00	7.25	2.00
Macromolecule binding	3.89	2.93	4.03	3.26	7.39	5.75
Ligand binding	9.74	4.65	9.55	4.97	13.72	7.06
Allosteric sites	1.49	1.00	1.74	1.00	3.16	2.00
All	13.28	8.35	12.93	9.21	17.43	13.36
*II. Multiple mechanisms*						
Confident biological hypotheses (*p*-value < 8.62 ⋅ 10^−4^)						
Metal binding	0.72	0.16	0.49	0.10	1.23	0.27
Macromolecule binding	0.05	0.04	0.17	0.04	0.22	0.07
Ligand binding	0.24	0.07	0.33	0.09	0.68	0.19
All	1.43	0.34	1.29	0.31	2.78	0.68
Actionable biological hypotheses (*p*-value < 0.01)						
Metal binding	5.96	2.31	4.75	2.42	10.37	4.62
Macromolecule binding	0.66	0.51	0.99	0.37	1.72	0.89
Ligand binding	5.55	1.90	5.43	1.85	11.04	4.11
All	12.88	5.42	12.22	5.42	23.57	10.60

For each functional site category, we show the relative contributions (%) of disease and neutral substitutions where at least one function *f* within a category has a greater *P*(loss|*x*)or *P*(gain|*x*) than a conservative threshold at 1% FPR. This threshold is estimated from the null distributions of *P*(loss|*x*) and *P*(gain|*x*) on the putatively neutral polymorphisms data set, respectively. The table is subdivided into two parts: (i) exactly one function (or mechanism) and (ii) two or more mechanisms. In both parts, the relative contributions are assessed at two p-value cutoffs of *p* < 8.62 ⋅ 10^−4^ and *p* < 0.01. Note that in a small number of cases, a loss of one function might result in the gain of another; thus, the sets of residues counted in the loss and gain may overlap.

Table 4 also shows situations with two or more functional perturbations consequent to the replacement of a given amino acid residue. The amino acid substitutions disrupting multiple functions may be important in a therapeutic context because addressing a single deficiency (e.g. iron binding) may still not result in a fully corrected phenotype because other deficiencies may still remain (e.g. ligand binding). Here, we have a significantly increased likelihood of observing multi-functional alterations in the disease set compared to the putatively neutral set; i.e., the disease set is several times more likely to contain multi-functional alterations than the putatively neutral set. For instance, 1.23% of disease mutations are likely to affect at least two metal binding sites versus only 0.27% of neutral variants, whereas 0.68% of disease variants may affect more than one ligand binding site as opposed to 0.19% of neutral polymorphisms.

**Fig 3:**
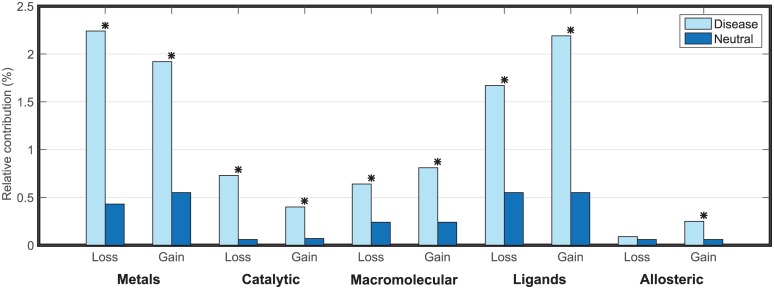
Relative contribution of loss and gain of functional categories on each amino acid substitutions data set. For each functional site category, we show the relative contributions (%) of disease and neutral variants where at least one function *f* within a category has a greater *P*(loss|*x*) or *P*(gain|*x*) than a conservative threshold at 1% FPR. This threshold is estimated from the null distribution of *P*(loss|*x*) and *P*(gain|*x*) on the putatively neutral polymorphisms data set, respectively. *Indicates significant *p*-value measured as a Fisher’s Exact test after Bonferroni correction for multiple hypothesis comparisons (*p* < 8.62⋅10^−4^).

If we combine the results for single and multiple mechanisms, we observe that 2.24% of disease variants are predicted, with high confidence, to impair metal-binding sites (1.51% loss of single site and 0.72% loss of multiple sites) and 1.67% probably impair ligand binding sites (1.43% loss of single site and 0.24% loss of more than one site), as depicted in Fig 3. Overall, we believe we can confidently propose molecular mechanisms of disease for 8.6% of all variants in the inherited disease data set whereas we only see about 2.4% of such variants in the neutral set. If we use a *p*-value cutoff of 0.01 without a Bonferroni correction, then we can computationally hypothesize a molecular mechanism for approximately 40.9% of disease variants.

### Validation of loss of function predictions

In this study, we have proposed a novel methodology for identifying specific molecular alterations of disease mutations. Given that it is impractical to experimentally validate the predicted functional effects of each individual amino acid substitution, we use mutagenesis experimental data to independently assess the loss of functional site predictions, as shown in S5 Table. To the best of our knowledge, this is the first time a systematic assessment of computationally predicted disruptions of specific types of functional residues has been carried out in the published literature.

**Fig 4:**
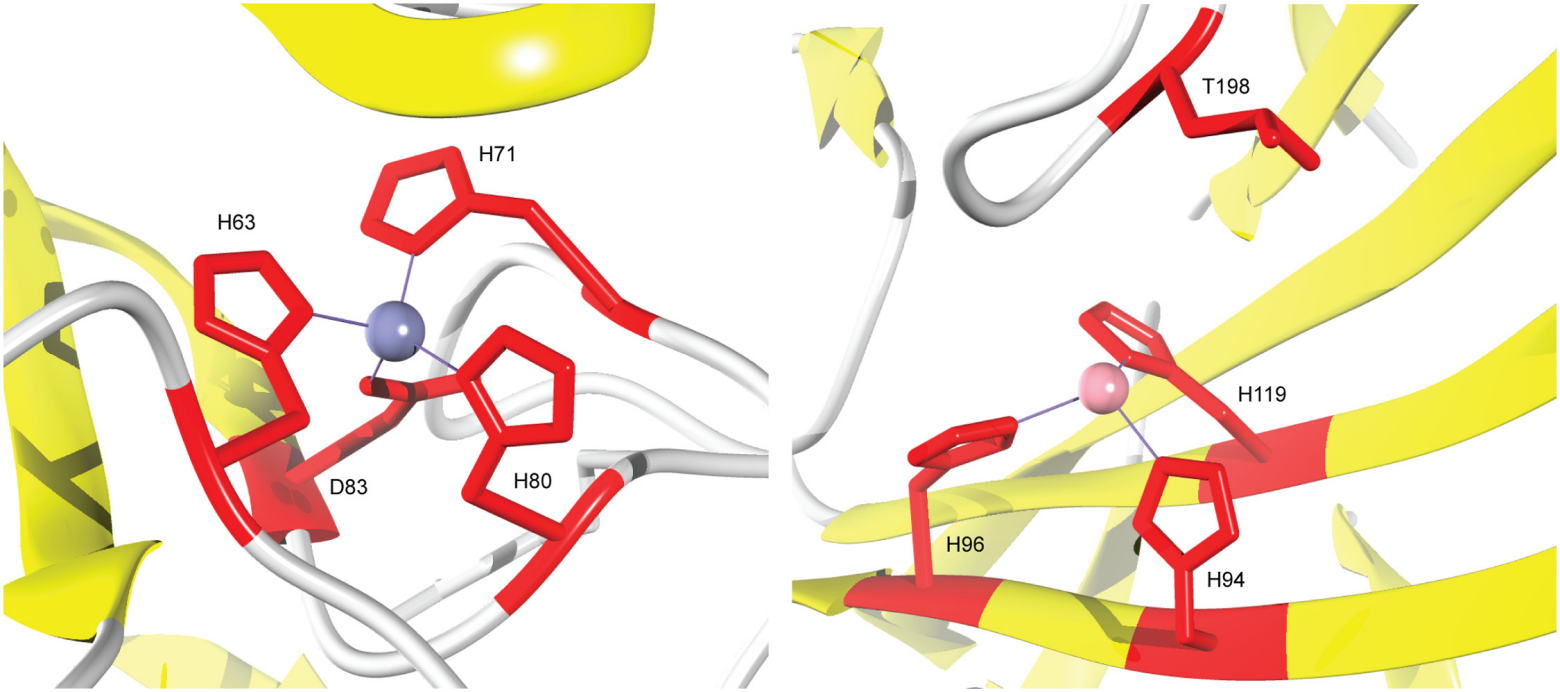
3D visualization of protein structures with experimentally supported loss and gain of function predictions. Left: SOD1 protein (chain A of PDB entry 2xjl) where residues H63, H71, H80 and D83 form a zinc binding pocket. The substitution D83G gives rise to a loss of zinc binding. Right: CA2 protein (chain A of PDB entry 1fqr) where H94, H96 and H119 are zinc-binding sites. Mutation T198E leads to an increase in zinc affinity.

In general, our loss of function predictors performed as expected. However, more interestingly, if one restricts the loss of function predictions to those with significant *p*-values (i.e. *p* < 0.01), then performance (AUC) rises to at least 95% for all predictors. This provides compelling evidence that our methodology can be effectively used to identify molecular mechanisms of disease and hence can be used to prioritize experimental validation. Additionally, Fig 4 depicts two case studies of loss and gain of function predictions which have been experimentally validated. We discuss each case in detail below:

#### Loss of zinc binding in superoxide dismutase (SOD1)

The functional role of SOD1 is to destroy radicals that are normally produced in cells and which are toxic to biological systems. SOD1 forms a zinc-binding pocket consisting of H63, H71, H80 and D83 [65, 66] as shown in Fig 4 (left). Mutations in SOD1 are known to be causative of amyotrophic lateral sclerosis [66–68]. However, the molecular mechanisms underlying these mutations often remain unclear. We predicted a loss of multiple functional activities for mutation D83G and identified zinc binding as the primary underlying molecular mechanism of disease. In particular, D83G has a $P(f|x_{wt}^{\prime })=0.99$ and $P\left(f|x_{mt}^{\prime }\right)=3.3\cdot 10^{-3}$ leading to a *P*(loss|*x*) ≈ 1, which is above the 1% FPR threshold of 0.20 with an empirical *p*-value of 1.2⋅10^−3^. A literature search for experimental evidence reveals that mutation D83G causes the destabilization of native structure which leads to protein aggregation with the formation of amyloid-like fibrils, and, ultimately, a gain of toxicity [69]. Zinc binding is a known stabilizer of protein structure and, therefore, the loss of the zinc-binding residue D83 appears to be a plausible destabilizing mechanism that ultimately impacts the biological function of SOD1. We note that the quadruple (H63, H71, H80, D83) was not part of the training data for the zinc-binding predictor.

This example raises an interesting possibility that the loss of a functionally important residue (zinc-binding residue) results in a loss of stability, and ultimately leads to disease through the loss of the protein’s function. In other words, protein structure and function appears to be intimately and bidirectionally interconnected. At this moment, however, this is only a theoretical possibility because of the lack of data about the structure and stability of the wild-type and mutant proteins in the absence of zinc ions.

#### Gain of zinc affinity in carbonic anhydrase 2 (CA2)

CA2 is essential for bone resorption and osteoclast differentiation. CA2 has three zinc-binding residues at H94, H96 and H119 as shown in Fig 4 (right). There are multiple studies that have characterized the effects of variants in CA2 via mutagenesis experiments [70–74]. Among these mutations, we predicted a gain of zinc binding for T198E that was experimentally shown to increase zinc affinity. Specifically, T198E has a $P\left(f|x_{wt}^{\prime }\right)=3.8\cdot 10^{-4}$ and $P(f|x_{mt}^{\prime })=0.99$ leading to a *P*(gain|*x*)≈1, which is above the 1% FPR threshold of 0.35 with a *p*-value of 3.7⋅10^−4^. The triple (H94, H96, H119) was not part of the training data for the zinc-binding residue predictor.

## Discussion

This study builds on the extensive prior work in structural bioinformatics to provide statistical evidence of the important role that alterations of multiple types of functional residue play in human genetic disease. Most of the existing work has centered around understanding the impact of sequence variants on protein stability or has only considered single types of function such as catalytic residues or protein-interaction sites [7, 20, 23, 24, 30, 75–78]. This work extends these studies by integrating the stability models with a series of functional residue predictors involving metal binding, macromolecular binding, ligand binding and others. Overall, we show and validate the feasibility of computationally predicting mutations that impair specific function using protein 3D structure data.

Despite using sophisticated methodology to model loss and gain of functional residues, the nature of this research has limitations involving both data sets and methodology. First, despite major efforts employed by authors and database curators when annotating amino acid substitutions as being causative of a particular disease, it is possible that some amino acid substitutions have been misannotated as disease-causing by the original authors reporting them. Similarly, mutagenesis experimental data are known to be biased toward certain amino acid residues. For example, alanine mutations comprised about 50% of the independent amino acid substitutions data set (due to the frequent use of alanine-scanning mutagenesis). There are also limitations and biases in relation to the protein structures available in PDB as well as in selecting an appropriate set of unlabeled variants. Second, there exist both theoretical and practical limitations in the semi-supervised framework used in this work. The accuracy of our methods is predicated upon the assumption that the computational models are capable of accurately estimating the posterior probability of the class labels. This however could not be guaranteed and thus requires caution when interpreting our results. Furthermore, there are identifiability issues in estimating class priors in the positive-unlabeled framework; i.e., the estimates for the class priors do not have a unique solution and only an upper bound can be estimated [27]. On the practical side, we have been careful to prevent overfitting. We performed only minor parameter selection steps before the final functional predictors were built. Thus, there is the potential to further improve predictor performance through more extensive work. This includes the use of additional features, optimizing the distance threshold used to define an edge between two residues when constructing protein contact graphs, choice of the capacity parameter in SVM^*light*^, among others. Finally, this work was designed to probabilistically reason about molecular mechanisms of disease and not necessarily to develop classifiers that outperform specialized models across the board. If a user needs a tool for a particular prediction task, we recommend that the most accurate predictor for this task be selected.

Despite these limitations, we believe this work contributes to an improved understanding of the impact of sequence variants on protein function. We have provided a model that considers functional alteration both when stability of the protein is disrupted and when it is not disrupted (e.g. interestingly, sequence changes can exert a functional effect in disordered regions such as disorder-to-order transition [79]). We believe that our work suggests a new class of approaches to disease studies that might qualify as mechanism-driven and disease-agnostic, where one might be compelled to identify a set of molecular alterations underlying a disease phenotype without necessarily studying a single disease. While each molecular alteration is likely to require an individualized approach to drug design and therapy, we envisage that the next generation of researchers might decide to specialize in addressing particular types of functional deficiencies rather than beginning with a particular disease.

## Supporting Information

S1 Fig Inverse cumulative distribution function (CDF) of *P*(loss|*x*). For ATP-binding predictor, we plot the inverse CDF for *P*(loss|*x*) on the disease and putatively neutral data sets, respectively.
(EPS)

S2 Fig Inverse cumulative distribution function (CDF) of *P*(gain|*x*). For catalytic residue predictor, we plot the inverse CDF for *P*(gain|*x*) on the disease and putatively neutral data sets, respectively.
(EPS)

S1 Table Mapping between ligand codes and names. (PDF)

S2 TableSelected kernel matrix parameters for each structural and functional site predictor.For each data set, we show the best-performing kernel matrix parameters obtained through a per-chain 10-fold cross-validation. The normalized edit distance kernel *k*_*m*_(*u*, *v*) outperformed both $k_{m}^{l}\left(u,v\right)$ and $k_{m}^{e}\left(u,v\right)$ on each data set. Note that the edit distance kernel with *m* = 0 is equivalent to a standard graphlet kernel.(PDF)

S3 Table Performance assessment of functional residue predictors using an independent data set. For each prediction method, we show number of proteins (*n*_*p*_), number of positive examples (*n*_+_), number of unlabeled examples (*n*_*u*_), AUC and sensitivity (*sn*) and MCC at score threshold corresponding to specificity (*sp*) of 99%. In the case of N-linked glycosylation (Nglyco), we only predict if the wild-type residue is an asparagine, whereas for phosphorylation (Phos), we only make predictions on threonine, tyrosine or serine residues.
(PDF)

S4 Table Class priors for structural and functional predictors. Fraction of residues in a data set estimated to be stability-impacting or functional. Estimates on the unlabeled data were made using the AlphaMax algorithm [27]; minor manual adjustments were made by observing the log-likelihood plots. *Indicates a confident prior estimate assessed by manually observing log-likelihood plots. Estimates on the disease and putatively neutral data were made using the empirical mean formula.
(PDF)

S5 TablePerformance assessment of loss of function predictions using mutagenesis experimental data.Independent validation of predicted loss of functional site events using a set of mutagenesis experimental data mapped to protein structures in PDB. This mutagenesis data set contains 3,356 AAS from 880 human proteins. For each functional feature, we show the number of experimentally determined losses (*n*_*l*_), AUC, sensitivity (*sn*) and MCC corresponding to a 99% specificity (*sp*) threshold. Additionally, the last five columns show the number of statistically significant (*p* < 0.01) loss-of-function predictions ($n_{l}^{*}$), as well as estimates for AUC (AUC*), sensitivity (sn*), specificity (sp*) and MCC (MCC*) on this filtered set.(PDF)
