# Supplementary material for: The Loss and Gain of Functional Amino Acid Residues Is a Common Mechanism Causing Human Inherited Disease
Source: PLoS Comput Biol. 2016 Aug 26;12(8):e1005091. doi: 10.1371/journal.pcbi.1005091 (PMC5001644; doi:10.1371/journal.pcbi.1005091)
Supplement: S1 Table — (PDF) [file pcbi.1005091.s003.pdf]

S1 Table

ADP	adenosine-5'-diphosphate
ATP	adenosine-5'-triphosphate
FAD	flavin-adenine-dinucleotide
FMN	flavin-mononucleotide
GDP	guanosine-5'-diphosphate
GTP	guanosine-5'-triphosphate
HEM	protoporphyrin-IX-containing-Fe
NAD	nicotinamide-adenine-dinucleotide
PLP	pyridoxal-5'-phosphate
UDP	uridine-5'-diphosphate
