# Supplementary material for: The Loss and Gain of Functional Amino Acid Residues Is a Common Mechanism Causing Human Inherited Disease
Source: PLoS Comput Biol. 2016 Aug 26;12(8):e1005091. doi: 10.1371/journal.pcbi.1005091 (PMC5001644; doi:10.1371/journal.pcbi.1005091)
Supplement: S2 Table — For each data set, we show the best-performing kernel matrix parameters obtained through a per-chain 10-fold cross-validation. The normalized edit distance kernel km(u, v) outperformed both kml(u,v) and kme(u,v) on each data set. Note that the edit distance kernel with m = 0 is equivalent to a standard graphlet kernel. (PDF) [file pcbi.1005091.s004.pdf]

S2 Table

Category	Site type	Kernel parameters
Protein stability	S	$m = 0, N = 4, \|\Sigma\| = 20$
Metal binding	Ca	$m = 1, N = 4, \|\Sigma\| = 20$
	Cd	$m = 1, N = 4, \|\Sigma\| = 20$
	Co	$m = 1, N = 4, \|\Sigma\| = 20$
	Cu	$m = 1, N = 4, \|\Sigma\| = 20$
	Fe	$m = 1, N = 4, \|\Sigma\| = 20$
	K	$m = 1, N = 4, \|\Sigma\| = 20$
	Mg	$m = 1, N = 4, \|\Sigma\| = 20$
	Mn	$m = 1, N = 4, \|\Sigma\| = 20$
	Na	$m = 1, N = 4, \|\Sigma\| = 20$
	Ni	$m = 1, N = 4, \|\Sigma\| = 20$
	Zn	$m = 1, N = 4, \|\Sigma\| = 20$
PTMs	Nglyco	$m = 0, N = 4, \|\Sigma\| = 20$
	Phos	$m = 0, N = 4, \|\Sigma\| = 20$
Catalytic sites	Cat	$m = 1, N = 4, \|\Sigma\| = 40$
Macromolecular binding	DNA	$m = 1, N = 4, \|\Sigma\| = 20$
	RNA	$m = 1, N = 4, \|\Sigma\| = 20$
	PPI	$m = 1, N = 4, \|\Sigma\| = 40$
	Hotspot	$m = 1, N = 4, \|\Sigma\| = 20$
Ligand binding	ADP	$m = 1, N = 4, \|\Sigma\| = 40$
	ATP	$m = 1, N = 4, \|\Sigma\| = 40$
	FAD	$m = 1, N = 4, \|\Sigma\| = 20$
	FMN	$m = 1, N = 4, \|\Sigma\| = 40$
	GDP	$m = 1, N = 4, \|\Sigma\| = 40$
	GTP	$m = 1, N = 4, \|\Sigma\| = 40$
	HEM	$m = 1, N = 4, \|\Sigma\| = 20$
	NAD	$m = 1, N = 4, \|\Sigma\| = 20$
	PLP	$m = 1, N = 4, \|\Sigma\| = 40$
	UDP	$m = 1, N = 4, \|\Sigma\| = 20$
Allosteric regulation	Allo	$m = 1, N = 4, \|\Sigma\| = 40$
