# Supplementary material for: The Loss and Gain of Functional Amino Acid Residues Is a Common Mechanism Causing Human Inherited Disease
Source: PLoS Comput Biol. 2016 Aug 26;12(8):e1005091. doi: 10.1371/journal.pcbi.1005091 (PMC5001644; doi:10.1371/journal.pcbi.1005091)
Supplement: S3 Table — For each prediction method, we show number of proteins (np), number of positive examples (n+), number of unlabeled examples (nu), AUC and sensitivity (sn) and MCC at score threshold corresponding to specificity (sp) of 99%. In the case of N-linked glycosylation (Nglyco), we only predict if the wild-type residue is an asparagine, whereas for phosphorylation (Phos), we only make predictions on threonine, tyrosine or serine residues. (PDF) [file pcbi.1005091.s005.pdf]

S3 Table

Category	Site type	n_p	n_+	n_u	AUC	sn	sp	MCC
Metal binding	Ca	816	17	2783	0.945	0.294	0.985	0.171
	Fe	817	26	2775	0.903	0.179	0.985	0.128
	Mg	817	32	2774	0.926	0.156	0.986	0.120
	Zn	817	106	2698	0.849	0.500	0.965	0.398
PTMs	Nglyco	89	30	86	0.902	0.400	0.988	0.539
	Phos	323	100	404	0.775	0.300	0.988	0.451
Catalytic sites	Cat	816	186	2616	0.860	0.255	0.965	0.254
Macromolecular binding	DNA	817	22	2787	0.739	0.091	0.986	0.057
	RNA	817	18	2791	0.682	0.056	0.986	0.028
	PPI	817	192	2610	0.558	0.063	0.965	0.037
Ligand binding	ATP	817	112	2694	0.848	0.159	0.985	0.200
	GTP	817	61	2748	0.749	0.180	0.987	0.188
	NAD	817	12	2795	0.891	0.083	0.985	0.037
