# Supplementary material for: The Loss and Gain of Functional Amino Acid Residues Is a Common Mechanism Causing Human Inherited Disease
Source: PLoS Comput Biol. 2016 Aug 26;12(8):e1005091. doi: 10.1371/journal.pcbi.1005091 (PMC5001644; doi:10.1371/journal.pcbi.1005091)
Supplement: S4 Table — Fraction of residues in a data set estimated to be stability-impacting or functional. Estimates on the unlabeled data were made using the AlphaMax algorithm [27]; minor manual adjustments were made by observing the log-likelihood plots. *Indicates a confident prior estimate assessed by manually observing log-likelihood plots. Estimates on the disease and putatively neutral data were made using the empirical mean formula. (PDF) [file pcbi.1005091.s006.pdf]

S4 Table

Data set	Class priors (%)		
	Unlabeled	Disease	Putatively neutral
S	12.5	13.6	6.94
Ca	0.83*	4.14	1.97
Cd	1.32	1.52	1.35
Co	0.58*	0.99	0.52
Cu	0.40*	0.60	0.31
Fe	0.17*	0.56	0.12
K	1.20	1.26	1.16
Mg	0.40*	0.83	0.34
Mn	0.01	0.12	0.04
Na	3.62	3.77	3.46
Ni	0.83*	1.00	0.87
Zn	1.32*	2.47	0.99
Nglyco	13.3*	13.8	15.9
Phos	0.44*	0.46	0.58
Cat	3.16*	5.47	2.45
DNA	0.48*	0.93	0.74
RNA	0.36*	0.16	0.21
PPI	16.3	19.9	27.9
Hotspot	0.50*	0.43	0.58
ADP	2.09*	3.96	2.28
ATP	2.95	3.70	2.92
FAD	2.31	4.40	2.43
FMN	0.45	0.58	0.46
GDP	0.83*	1.13	0.84
GTP	1.22	1.37	1.37
HEM	2.76	3.94	3.15
NAD	2.06	3.26	1.98
PLP	0.72*	1.59	0.77
UDP	4.25	4.92	4.52
Allo	4.85	5.08	5.27
