# Supplementary material for: The Loss and Gain of Functional Amino Acid Residues Is a Common Mechanism Causing Human Inherited Disease
Source: PLoS Comput Biol. 2016 Aug 26;12(8):e1005091. doi: 10.1371/journal.pcbi.1005091 (PMC5001644; doi:10.1371/journal.pcbi.1005091)
Supplement: S5 Table — Independent validation of predicted loss of functional site events using a set of mutagenesis experimental data mapped to protein structures in PDB. This mutagenesis data set contains 3,356 AAS from 880 human proteins. For each functional feature, we show the number of experimentally determined losses (nl), AUC, sensitivity (sn) and MCC corresponding to a 99% specificity (sp) threshold. Additionally, the last five columns show the number of statistically significant (p < 0.01) loss-of-function predictions (nl*), as well as estimates for AUC (AUC*), sensitivity (sn*), specificity (sp*) and MCC (MCC*) on this filtered set. (PDF) [file pcbi.1005091.s007.pdf]

S5 Table

Category	Site type	n_l	AUC	sn	sp	MCC	n_l^*	AUC*	sn^*	sp^*	MCC*
Metal binding	Ca	13	0.858	0.077	0.985	0.032	2	0.990	1.00	0.985	0.139
	Fe	42	0.841	0.143	0.985	0.112	23	0.971	0.261	0.985	0.160
	Mg	35	0.877	0.114	0.985	0.081	24	0.958	0.167	0.985	0.103
	Zn	47	0.922	0.489	0.966	0.271	30	0.975	0.767	0.966	0.350
PTMs	Nglyco	11	0.841	0.727	0.791	0.322	2	0.985	0.500	0.992	0.492
	Phos	109	0.779	0.068	0.991	0.144	23	0.981	0.174	0.991	0.260
Catalytic activity	Cat	1226	0.706	0.045	0.985	0.091	204	0.978	0.270	0.985	0.383
Macromolecular binding	DNA	38	0.722	0.105	0.985	0.077	17	0.951	0.235	0.985	0.126
	RNA	52	0.546	0.039	0.988	0.029	1	1.00	1.00	0.988	0.153
	PPI	616	0.631	0.037	0.986	0.067	29	0.990	0.793	0.986	0.540
Ligand binding	ADP	12	0.685	0.083	0.986	0.034	2	0.989	0.500	0.986	0.099
	ATP	121	0.855	0.141	0.988	0.187	50	0.980	0.340	0.988	0.310
	GDP	17	0.543	0.177	0.985	0.092	3	0.995	1.00	0.985	0.236
	GTP	39	0.642	0.051	0.987	0.036	4	0.989	0.500	0.987	0.146
	NAD	16	0.768	0.063	0.985	0.027	7	0.981	0.143	0.985	0.048
